# Histological validation of ShMOLLI equilibrium contrast CMR for the measurement of diffuse myocardial fibrosis

**DOI:** 10.1186/1532-429X-14-S1-O111

**Published:** 2012-02-01

**Authors:** Steven K White, Stefan K Piechnik, Stefan Neubauer, Matthew D Robson, James Moon

**Affiliations:** 1 The Heart Hospital Imaging Centre, The Heart Hospital, London, UK; 2 The Hatter Cardiovascular Institute, University College London, London, UK; 3 Oxford Centre for Clinical Magnetic Resonance Research (OCMR), University of Oxford, Oxford, UK

## Background

Diffuse myocardial fibrosis can be measured using pre and post contrast T1 relaxation time changes. Newer, faster sequences for T1 mapping promise whole heart coverage and improved clinical utility, but have not been validated against histology.

## Methods

In fourteen patients with symptomatic severe aortic stenosis awaiting valve surgery, we performed equilibrium contrast CMR (EQ-CMR: [Flett AS et al. Circulation 2010;122(2):138-44]) to calculate Vd(m) using ShMOLLI (Shortened Modified Look-Locker Inversion recovery [Piechnik at al. JCMR 2010;12:69]) and standard multibreathold (FLASH) mapping, for the pre and equilibrium contrast T1 mapping. We compared the results to surgical biopsy.

Vd(m) was calculated by Vd(m)=(1-hematocrit)xΔ(1/T1)myo ÷ Δ(1/T1)blood.

Surgical left ventricular septal biopsies were fixed and stained with picrosirius red and then digitally photographed. Collagen volume fraction (CVF%) was calculated by a blinded observer using in-house software (macro written in Image J) for automated analysis. Patients with LGE in the biopsy area were pre-specified as being excluded from analysis.

## Results

FLASH T1 mapping was not possible in 2 out of 14 patients due to: 1) inability to breath hold & 2) persistent ectopy. ShMOLLI assessment was possible in all subjects. No patient was excluded for LGE in the biopsy area, but 2 biopsy specimens were excluded because they were thought histologically to be superficial with extremes of fibrosis (patchy fibrosis).

**Figure 1:**
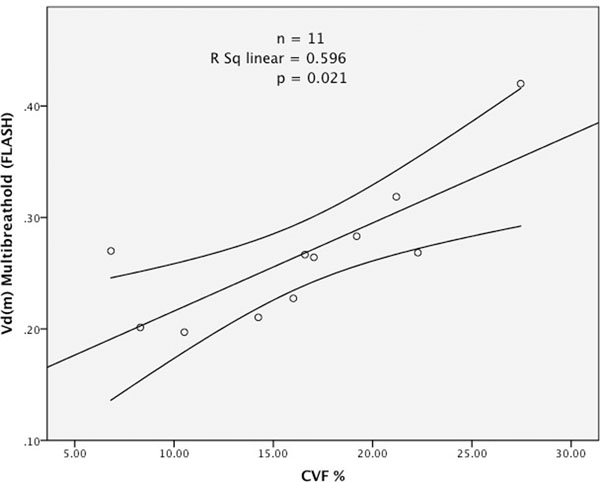
MRI measured myocardial volume of distribution Vd(m) against histological CVF% (n=11, r=0.772, p=0.021). Vd(m) calculated using T1 values obtained by Inversion Recovery FLASH acquired over multiple breath-holds with different TI delays.

**Figure 2:**
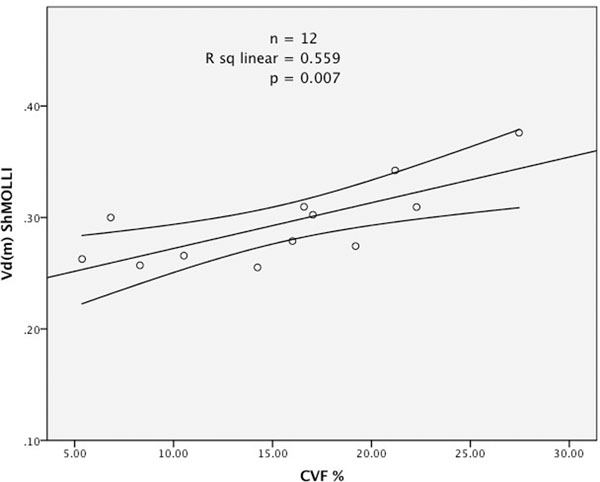
MRI measured myocardial volume of distribution Vd(m) against histological CVF% (n=12, r= 0.748, p=0.007). Vd(m) calculated using T1 values obtained by ShMOLLI.

There was a strong correlation between histological CVF% and both FLASH Vd(m) (r=0.772, p=0.021) and ShMOLLI Vd(m) (r=0.748, p=0.007), as shown in Figures 1 and 2.

## Conclusions

Rapid T1 mapping with ShMOLLI can be used to measure Vd(m) by EQ-CMR. The histological calibration here permits conversion of ShMOLLI Vd(m) to % fibrosis, but also, potentially, whole heart fibrosis assessment, and Vd(m) in patients unsuitable for slower, mutibreathold, mapping techniques.

## Funding

SKW is funded by the British Heart Foundation.

SKP and MDR are funded by the NIHR Oxford Biomedical Research Centre Programme.

